# Low-cost solar power enables a sustainable energy industry system

**DOI:** 10.1073/pnas.2116940118

**Published:** 2021-11-29

**Authors:** Christian Breyer

**Affiliations:** ^a^School of Energy Systems, LUT University, Lappeenranta 53850, Finland

Hothouse Earth is endangering the stability of planetary ecosystems at an unprecedented level; consequently, an immediate phase-out of anthropogenic greenhouse gases is required to restabilize global ecosystems (1). Approximately 84% of global CO_2_ emissions are anthropogenic, relating to the energy system and industrial activities (2). The leading economies of the world must, therefore, assume a role of true global leadership in order to develop a strategy for the survival of mankind. In PNAS, Lu et al. (3) show, with analytical precision, how China could not only massively reduce CO_2_ emissions but also further boost its economic growth from CO_2_ reduction through the utilization of low-cost electricity. Because solar photovoltaic (PV) electricity will be cheaper than China’s current coal-based electricity supply, China can expect substantial economic growth from a massive and rapid ramping up of solar PV capacities. The solar PV potential in China is several factors larger than its long-term total energy demand, as the 99.2 PWh of identified technical potential as of 2020 is projected to increase to 146.1 PWh in 2060. As of 2021, the current technical potential of 78.2% of the 2020 value has already reached price parity with coal-based electricity generation, according to Lu et al. (3). Since the execution of this research, coal prices have reached even higher levels. The steep cost decline of solar PV is a catalyst for the integration of other energy technologies required for a highly sustainable energy system, in particular, battery storage and electrolyzers. Consequently, this leads to the fundamental conclusion of Lu et al. (3) that PV battery systems will form a central pillar of the power system in China due to the system’s low cost, sustainability, scalability, and distributed resource availability across the entire country.

## Role of Low-Cost Solar PV for China, India, and Worldwide

The contribution from Lu et al. (3) presents the most detailed technoeconomic analysis to date of the solar P V potential in China. By considering the latest cost developments of solar PV and battery storage, the outstanding competitiveness of solar PV is documented for the present, and this cost attractiveness is projected to further increase during the next few decades. Several studies on highly renewable energy systems for China have demonstrated the technical feasibility and the economic viability of PV battery systems both outside (4–6) and inside (7, 8) China. The cobenefits of an accelerated ramping of PV capacity are even higher than what is found in most energy system analyses. Typically, the huge cost of coal-induced air pollution is neglected, while, conversely, the additional jobs created by PV battery systems (9) trigger additional societal benefits. The overall societal benefits of an accelerated PV battery capacity increase may lead decision makers to develop even stronger policies for a rapid energy transition in the years to come. Finally, a low-cost energy supply is the foundation for international economic competitiveness; therefore, from the early 2020s onward, a concentrated focus must be placed on renewable energy and, in particular, solar PV.

The role of solar PV in the low-cost and fast transition of an energy system has been strongly underestimated, not only in the scientific literature but also by most major institutions (10–12). Lu et al. (3) find a solar PV share of 43.2% in the power supply of China in 2060, which is one of the highest values ever projected. Only Bogdanov et al. (6) present a cost-optimized power sector analysis for China with an even higher share of 70.5% in 2050. Indeed, PV battery systems emerge as a central pillar of a low-cost and sustainable power system, as found by Lu et al. (3) for China, and by Gulagi et al. (13) for India. The geography of India, being farther south, enables an even higher solar PV supply share of 89% in its power sector. This result is a consequence of both higher solar PV potential and lower potential of wind energy and hydropower compared to China. Interestingly, the seasonal variation in China is mainly managed with the complementarity of wind and solar resources, while, for the case of India, the monsoon period can be also managed by solar PV–based electricity supply through transmission grids. For various European countries, being located farther north than China, PV shares higher than 50% have been projected (14). Thus, the ever-increasing number of energy system studies identifying very high shares of solar PV in energy supply around the world (14) indicates the strong economic competitiveness of solar PV. Furthermore, these findings suggest that the rapid cost decline of solar PV has been increasingly implemented in energy system analyses. In total, 34 publications for countries in all continents are known with PV shares of at least 50% for a sustainable energy system configuration by midcentury (14). Nevertheless, substantial deficits in awareness of real low-cost sustainable energy system solutions exist (10–12), and Lu et al. (3) contribute to overcome these outdated views.

## Value of Low-Cost Solar PV for the Entire Energy System

Very low-cost solar PV disrupts not only the power sector but the entire energy system as well (15, 16). The transition from a fossil fuel–based energy system to that based on renewable electricity suggests that combustion processes utilizing heat will be substituted by direct electricity–based solutions wherever possible as well as indirect use of electricity with power-to-X processes, in particular for electricity-based e-fuels, e-chemicals, and green steel. Various power-to-steel options are known (17), and green hydrogen-based steelmaking is currently prepared to phase out coal-based steelmaking. Green methanol is expected to become a new major bulk chemical, substituting for fossil feedstock (18). Green ammonia is expected to substitute for fossil fuel–based ammonia (19), primarily as a feedstock for fertilizers, although it could be also used for energy applications. E-fuels for marine and aviation (16, 20) are required to defossilize long-distance transportation. What do green steel, green e-chemicals, and e-fuels have in common? They each require very low-cost and sustainable electricity for green hydrogen production as the first step of power-to-X conversion. As the least-cost source of electricity generation of newly installed capacities, solar PV electricity emerges as the primary electricity source for power-to-X processes in most regions around the world. At present, fossil methane and coal market prices are 80 € per MWh_NaturalGas_ and more than US$200 per ton of coal, respectively, and green ammonia based on hybrid PV–wind power plants (19) is the lower-cost option, now. Green ammonia is the first e-chemical/e-fuel that is cost competitive with the fossil solutions of the present, indicating that the other e-fuels and e-chemicals will follow in the near term to midterm. Thus, solar PV will disrupt not only the power sector but the entire energy industry system (14, 16).

## Requirement for Scaling PV Industry

The major challenge ahead will be to scale the global solar PV manufacturing from the newly installed PV capacity of 138.5 gigawatt-peak (GWp) in 2020 to the more than 1 terawatt-peak (TWp) needed at the end of the 2020s and about 3 TWp in the 2030s (21). Achieving these targets requires massive industrial scaling for delivering the required PV capacity so that low-cost PV electricity can enable energy supply in the power sector, direct electrification of heat and transportation, and indirect electrification via power-to-X. Several researchers have indicated that multi-TWp annual PV manufacturing is possible (10, 15, 21, 22). However, some adjustments are required to avoid bottlenecks in scaling the PV industry, such as switching from silver-based contacts in solar cells to copper-based solutions, comprehensive circular economy approaches with the highest standards in recycling, and a fast scaling of the glass industry. An optimized industrial scaling seems to be achievable with continued annual manufacturing output growth of 25 to 30% per year for the entire 2020s and early 2030s (21).

## Net-Negative CO_2_ Emissions Enabled by Low-Cost Solar PV

The long-term solar PV demand as a major energy supply for mankind may reach 170 TWp by the end of the century as independently projected by Goldschmidt et al. (22) and Breyer et al. (14). A very low-cost and sustainable solar PV electricity supply based on abundant materials may enable an unprecedented level of human development with energy wealth for all, while simultaneously enabling a global temperature stabilization at 1.5 °C or even below. Direct air captured CO_2_ and storage (DACCS) may act as a scalable and comparably low-cost net-negative CO_2_ emission option (14), which can be fully based on solar PV electricity supply. The main carbon dioxide removal (CDR) options are afforestation, bioenergy CCS, and DACCS (14), while the latter may be the best scalable and most economically viable solution. Similar to solar PV, batteries, and electrolyzers, CO_2_ direct air capture (DAC) units are highly modular and thus follow similar cost reduction trends, leading to attractive economics of capturing CO_2_ from the atmosphere. CO_2_ DAC is not yet as mature as solar PV, batteries, and electrolyzers, as its commercialization has only recently been started in projects in Chile, Iceland, and elsewhere. The status and role of CO_2_ DAC is a matter of discussion (23), although a very high potential for DACCS has been known for about a decade (24). Similarly known is that large-scale CDR will be required for achieving climate safety, as Hansen et al. (25) have suggested a rebalancing at 350 ppm of atmospheric CO_2_. Based on this climate science recommendation, initial estimates revealed that not more than 10% of the planned global PV capacity for energy and industry demands has to be added for PV-based DACCS net-negative CO_2_ emissions facilities to rebalance at 350 ppm by the end of this century. Applying high shares of the other CDR options may reduce the demand for PV-based DACCS and mitigate scaling risks, although these options may have much smaller sustainable CO_2_ removal potentials. More scientific discourse will be required for a comprehensive investigation of such an energy industry CDR system, mainly based on solar PV.

### The contribution from Lu et al. presents the most detailed technoeconomic analysis to date of the solar PV potential in China.

Lu et al. (3) find an outstanding cost attractiveness for solar PV in China for today, which had not been imaginable a few years ago. These findings for China, as a country of the Global North, indicate an even higher role of solar PV for countries of the Global South. The disruption of the power sector with low-cost solar PV electricity will be followed by a substantial solar PV share in the primary energy supply for the entire energy system, for chemical feedstock, and even as a major energy supply for net-negative CO_2_ emissions solutions needed for keeping the 1.5 °C target of the Paris Agreement. China can lead the sustainable energy transition with developing solutions that can be used as a blueprint for most countries all around the world.
